# A Randomized Pilot Trial Comparing Position Emission Tomography (PET)-Guided Dose Escalation Radiotherapy to Conventional Radiotherapy in Chemoradiotherapy Treatment of Locally Advanced Nasopharyngeal Carcinoma

**DOI:** 10.1371/journal.pone.0124018

**Published:** 2015-04-27

**Authors:** Jianshe Wang, Junnian Zheng, Tianyou Tang, Feng Zhu, Yuanhu Yao, Jing Xu, Andrew Z. Wang, Longzhen Zhang

**Affiliations:** 1 Department of Radiation Oncology, Affiliated Hospital of Xuzhou Medical College; Cancer Institute of Xuzhou Medical College, Jiangsu, China; 2 Department of Radiation Oncology, University of North Carolina-Chapel Hill, Chapel Hill, North Carolina, United States of America; 3 PET-CT Center Xuzhou Central Hospital, Xuzhou, Jiangsu, China; Colorado State University, UNITED STATES

## Abstract

**Background:**

This pilot trial is designed to determine whether PET/CT-guided radiotherapy dose escalation can improve local control while minimizing toxicity for the treatment of locally advanced nasopharyngeal carcinoma.

**Methods:**

67 patients were randomized into the three treatment arms: conventional chemoradiotherapy (group A), CT-guided dose escalation chemoradiotherapy (group B) and PET/CT-guided dose escalation chemoradiotherapy (group C). Radiotherapy was delivered using the simultaneous modulated accelerated radiation therapy (SMART) technique in the dose-escalation treatment arms. Patients received concurrent and adjuvant chemotherapy.

**Results:**

The use of PET/CT significantly changed the treatment volume delineation of the gross tumor volume. 3-year local progression-free (LPF) survival rates of three groups were 83.3%, 90.9% and 100%, respectively. The 3-year regional progression-free survival (RPFS) rates were 95.8%, 95.5% and 100%, respectively. The 3-year disease free survival (DFS) rates were 79.2%, 86.4% and 95.2%, respectively. The 3-year overall survival (OS) rates were 83.3%, 90.9% and 95.2%, respectively. The 3-year disease-free survival (DFS) rates were 79.2%, 86.4% and 95.2%, respectively. No patient had grade 4 late toxicity.

**Conclusions:**

PET/CT-guided dose escalation radiotherapy is well-tolerated and appears to be superior to conventional chemoradiotherapy for locally advanced NPC.

**Trial Registration:**

ClinicalTrials.gov NCT02089204

## Introduction

Nasopharyngeal carcinoma (NPC) differs from other head and neck malignancies in terms of its epidemiology, pathology, and treatment outcomes [1]. It is endemic in China and is one of the major public health problems. Concurrent radiotherapy and chemotherapy is the primary treatment for patients with NPC [2]. Despite such aggressive treatment, many patients with locally advanced NPC still develop locally recurrent disease [3]. Since local control is directly related to patient morbidity and mortality in NPC, there is a strong need to identify methods to further improve treatment outcome for NPC.

One strategy to improve local control is to escalate the dose of radiotherapy. This is because local control has been shown to be directly related to the radiotherapy dose [4,5]. Several different techniques, including brachytherapy [6], stereotactic radiosurgery [7], and dose-painting intensity modulated radiotherapy (IMRT) [8], have been used to increase radiotherapy dose. However, due to the large number of critical anatomic structures near the nasopharynx, dose-escalation in NPC can also lead to increased toxicities [9]. The main challenge for such a treatment is to identify the appropriate tumor volume to receive the high-dose radiotherapy. Conventional dose-escalation is conducted using computed tomography (CT) to identify the gross tumor volume (GTV). However, recent progress with fluorine-18-fluorode-oxyglucose positron emission tomography/computed tomography (^18^F-FDG-PET/CT) in treatment planning allows more accurate tumor volume delineation [10]. We hypothesize that the use of PET/CT in treatment planning can improve dose-escalation radiotherapy for NPC, which in turn can improve therapeutic efficacy while reducing toxicity. Given that there has been no clinical trials directly comparing conventional chemoradiotherapy to CT-guided dose-escalation chemoradiotherapy or PET/CT guided dose-escalation chemoradiotherapy in locally advanced NPC, our study aims to compare the local control, overall survival and toxicities of the three treatment regimens.

## Materials and Methods

### Study objectives and eligibility criteria

This study was approved by the Institutional Review Board of Xuzhou Medical School (S1 Checklist). All procedures were in accordance with the ethical standards of the responsible committee on human experimentation and with the Helsinki Declaration of 1975, as revised in 2008 (S1 Protocol). As a pilot trial, patients with previously untreated Stages III and IVA (AJCC 6th Edition) of locally advanced NPC, Karnofsky performance status≥70, and good bone marrow, liver and kidney function (white blood cell count ≥ 4.0×10^9^/L, platelets ≥ 100×10^9^/L, albumin ≥30 g/L, creatinine ≤100μmol/L) were enrolled on this study between February 2009 to March 2011. Written informed consent was obtained from each patient. Patients younger than 18, as well as those with a prior (within 5 years) or synchronous malignancy were excluded. Pretreatment evaluation consisted of a history and physical, dental and laboratory studies. The clinical stage was determined based on all information provided by examinations including contrast enhanced CT and magnetic resonance imaging (MRI) of head and neck, chest radiograph, liver sonography, bone scan, and ^18^F-FDG-PET. All tumors were histologically confirmed except those of distant metastases.

The primary objective of our study was to compare the local progression-free (LPF) survival rates of the three treatment regimens. Our secondary objectives were to compare the change in staging from PET imaging, regional progression-free survival (RFS), disease-free survival (DFS), overall survival (OS), and acute and late toxicities. When this trial was initiated, our institution did not register our clinical trials with international registries such as clinicaltrials.gov. Like other trials during the same enrollment period from our institution, this trial was not registered with clinicaltrials.gov until enrollment has completed. The infrastructure and support for trial registration has since been made available and all ongoing and related trials for this drug/intervention are registered.

### Study Design

**Fig 1:**
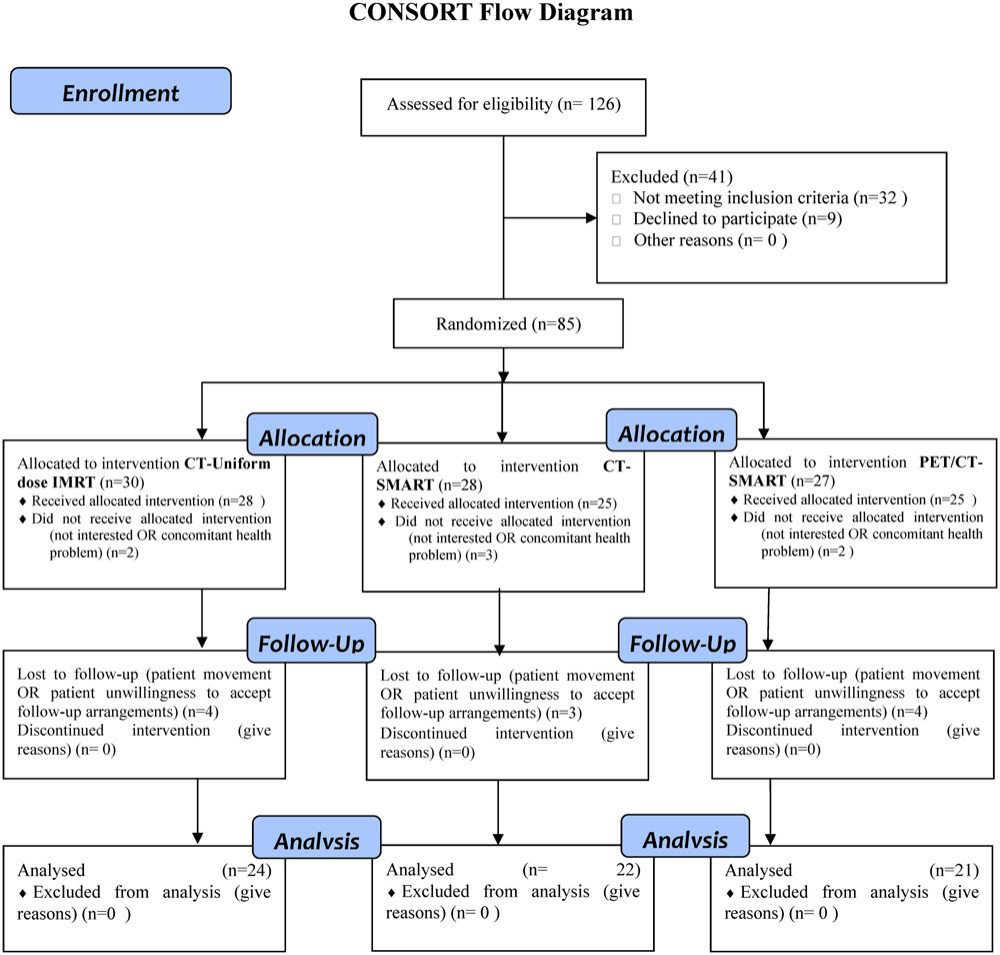
CONSORT flow diagram of trial.

Patients who met the eligibility criteria were randomized 1:1:1 into the three treatment arms: conventional chemoradiotherapy (group A), CT-guided dose escalation chemoradiotherapy (group B) and PET/CT-guided dose escalation chemoradiotherapy (group C). All patients were given concurrent chemoradiotherapy within two weeks of diagnosis (Fig 1).

For radiotherapy treatments, patients were immobilized in the supine position with a thermoplastic mask extending to the shoulders. In groups A and B, GTVs were delineated based on fusing diagnostic CT images with simulation CT images. For group C, images from a diagnostic PET/CT are fused to the treatment CT. Areas with standardized uptake value (SUV) ≥2.5 were used as the GTV [11,12]. Two clinical target volumes (CTVs) were delineated: CTV_1_ and CTV_2_. CTV_1_ was defined as GTV plus a 5 to 10 mm margin (2 to 3 mm margin posteriorly) to encompass the high-risk sites of microscopic extension (including the parapharyngeal spaces, posterior third of nasal cavities and maxillary sinuses, pterygoid processes, base of skull, lower half of sphenoid sinus, anterior half of clivus, and petrous tips), and the level of the lymph node located (bilateral levels IIa, IIb, III, and Va are routinely covered for all N_0_ patients, whereas ipsilateral levels IV, Vb, or supraclavicular fossae were also included for N_1_ patients). CTV_2_ was defined as the CTV_1_ plus a 5 to 10 mm margin (2 to 3 mm margin posteriorly) to encompass the low-risk sites of microscopic extension. The planning target volume (PTV) was defined as the CTV plus 3-mm margins. Patients in group A received radiotherapy delivered with the intensity modulated radiotherapy (IMRT) technique. PTV_1_ received 70 Gy in 2 Gy per fraction whereas PTV_2_ received 58 Gy in 2 Gy per fraction in groups A. For groups B and C, PTV_1_ received 63 Gy and PTV_2_ received 54 Gy, all in 1.8 Gy per fraction. In addition, the GTV received 70 Gy in 2.2 Gy per fraction in group B and the GTV received 77Gy in 2.4 Gy per fraction in group C. Radiotherapy was delivered using the SMART-IMRT technique in the dose-escalation treatment arms [13]. Parotid gland dose was limited to a mean dose below 30Gy [14] and the temporal lobes dose was limited to below 55Gy, both without compromising PTV or GTV coverage. Other dose constraints were used per RTOG 0225 protocol.

Concurrent chemotherapy consisted of cisplatin (20 mg/m^2^, IV, d1–4) and docetaxel (75 mg/m^2^, IV, d1 and d8) administered on the 1st and 4th week of treatment. All patients received adjuvant chemotherapy (starting 4 weeks after radiotherapy) of the same dose and drug regimen that ranged from 2 to 4 cycles.

### Follow-up and statistical analysis

Planned patient assessments included physical examination and fiberoptic nasopharyngoscopy every 3 months, starting at 4 weeks and ending 3 years post-treatment. A contrast-enhanced CT or MRI of the head and neck is also obtained at each follow up. After 3 years, the patients were followed yearly thereafter. Suspected recurrences were histologically proven. To assess for distant metastasis, CT of the chest and bone scan were obtained every 6 months. During every follow-up visit, treatment toxicity was assessed. Radiotherapy-related toxicities were graded according to the Acute and the Late Radiation Morbidity Scoring Criteria of the Radiation Therapy Oncology Group (RTOG) and the European Organization for Research and Treatment of Cancer (EORTC). Chemotherapy-related toxicities (except nausea or alopecia) were graded by the criteria of the WHO.

All events were measured from the date of randomization. The primary endpoint was local progression-free survival (LPFS), and the secondary endpoints were local control, regional progression-free survival (RPFS) disease free survival (DFS), overall survival (OS) and radiation toxicities. LPFS was defined as the time from the beginning of randomization to local progression of primary tumor area. RPFS was defined as the time from the beginning of randomization to progression of non-primary tumor within the treatment regions. Overall survival (OS) was defined as the time from the date of randomization to death or the latest date known to be alive and compared using Log-Rank test. The patient characteristics between the 3 groups were analyzed using Fisher’s exact test. The Kaplan-Meier method was used to calculate the rates of local control, regional control and DFS. Comparison of different treatment groups was performed using log-rank test. Univariate analysis was performed using the log-rank test. The Cox proportional hazard model was used in the multivariate comparisons, and an estimated hazard ratio (HR) with a 95% confidence interval (95% CI) was presented. All statistical analyses were two-sided and a p value of <0.05 was considered statistically significant. All analyses were performed using SPSS version 16.0 software (SPSS Inc., Chicago, IL, USA).

## Results

### Patient characteristics

67 eligible patients (43 male, 24 female) with a mean age of 47.5 years (range, 19–68 years) were enrolled in the study (S1 Table). Characteristics of the patients are outlined in Table 1. All patients successfully completed treatment within 7 weeks. The mean treatment time was 46 day (range, 44–49 day), 32 to 35 fractions. All patients received the prescription dose of radiotherapy and concurrent chemotherapy. Among the 63 patients who received adjuvant chemotherapy, the mean number of cycles received was 3. Of the 4 patients who did not receive adjuvant chemotherapy, 2 were from group C and 1 from Groups A and B each. These patients declined further treatment.

**Table 1:** Demographic and Clinical Characteristics of 67 Patients.

Patient characteristics	Group A	Group B	Group C	*P* value*
Patient number	24	22	21	
Gender				
Male	15	14	14	
Female	9	8	7	>0.05
Age (yr)				
Range	19~67	20~68	19~64	
Mean	47	48	46	>0.05
Clinical stages				
III stage	14	13	14	
IVa stage	10	9	7	>0.05
T stage				
T1	1	1	1	
T2	10	9	7	
T3	7	5	9	
T4	6	6	4	>0.05
N stage				
N0	1	1	1	
N1	3	4	3	
N2	15	14	13	
N3	5	3	4	>0.05
Pathologic types				
WHO II	5	4	3	
WHO III	19	18	18	>0.05

*Chi-square test performed.

Group A: conventional chemoradiotherapy group; Group B: CT-guided dose escalation chemoradiotherapy group; Group C: PET/CT-guided dose escalation chemoradiotherapy group; WHO: World Health Organization.

### The impact of PET/CT on staging and GTV

In group C, the staging of 3 patients (14.3%) was changed after PET/CT imaging. In one patient, PET/CT scan revealed tumor invading the orbit and base of skull as well as bilateral level II lymph nodes, whereas contrast CT did not. The patient’s clinical stage was upstaged from T3N0M0 to T4N2M0. Another patient was also upstaged from T3N2M0 to T3N3M0 due to identification of supraclavicular lymph node metastases on PET/CT. In the third patient, PET/CT identified a second primary tumor, papillary thyroid carcinoma, confirmed by biopsy. The patient underwent thyroidectomy after chemoradiotherapy for NPC.

To determine the impact of PET/CT on GTV delineation, GTV was drawn using both PET/CT and planning CT (with contrast). The change in GTV volume was calculated as (*GTV*_*PET—CT*_ *-GTV*_*CT*_)/*GTV*_*CT*_. The absolute value of volume change > 25% was defined as significant changes. 85.7% (18/21) patients’ GTV were smaller based on PET/CT. The GTV in 47.6% (10/21) of the patient had significant changes (Table 2). Complete data is included in S2 Table.

**Table 2:** The change in GTV volume in Group C patients based on PET/CT.

No	GTV_C T_(cm^3^)	GTV_PET-CT_(cm^3^)	Percent of changes(%)
*1*	*15*.*28*	*11*.*09*	*-27*.*42*
*2*	*7*.*43*	*4*.*12*	*-44*.*55*
*3*	*10*.*62*	*7*.*57*	*-28*.*72*
*4*	*30*.*15*	*21*.*72*	*-27*.*96*
5	*15*.*26*	*13*.*05*	*-14*.*48*
6	*32*.*41*	*29*.*27*	*-9*.*69*
*7*	*15*.*37*	*8*.*66*	*-43*.*66*
8	*44*.*13*	*55*.*17*	*25*.*02*
*9*	*4*.*52*	*2*.*77*	*-38*.*12*
*10*	*18*.*64*	*13*.*14*	*-29*.*51*
*11*	*19*.*85*	*14*.*68*	*-26*.*05*
12	*49*.*23*	*54*.*94*	*11*.*60*
*13*	*9*.*46*	*5*.*74*	*-39*.*32*
14	*28*.*31*	*22*.*07*	*-22*.*04*
15	*30*.*68*	*26*.*75*	*-12*.*81*
16	*34*.*16*	*28*.*25*	*-17*.*50*
17	*40*.*42*	*34*.*56*	*-14*.*50*
18	*42*.*15*	*47*.*36*	*11*.*00*
19	*22*.*38*	*17*.*34*	*-22*.*39*
20	*55*.*64*	*42*.*34*	*-23*.*80*
21	*31*.*75*	*26*.*42*	*-16*.*79*

### Local control and Survival

**Fig 2:**
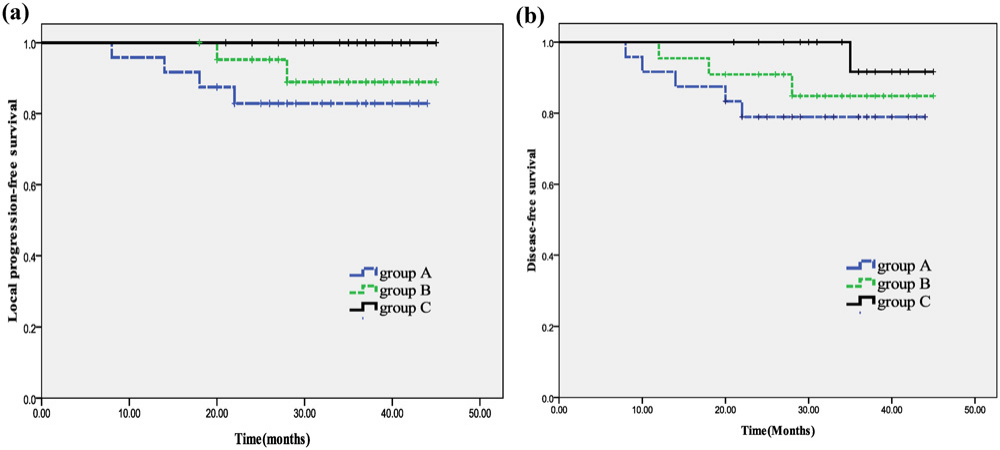
Kaplan-Meier survival analysis of the treatment groups. (a). The 3-year LPF survival rates of three groups. (b). The 3-year DFS rates of three groups. Group A: conventional chemoradiotherapy group; Group B: CT-guided dose escalation chemoradiotherapy group; Group C: PET/CT-guided dose escalation chemoradiotherapy group. The 3-year LPF and DFS were statistically significant between groups A and C (P <0.05).

The median follow-up was 36 months (range, 20–45 months). All patients were followed according to protocol. Group A had 4 patients (16.7%) who died from NPC (survival times were 10 months, 15 months, 18 months, and 22 months), and 4 patients (16.7%) who developed local recurrence (all with T4 disease, and with in-field failures in the GTV). 1 patient (4.2%) had regional recurrence and 5 patients (20.8%) developed distant metastasis (lung metastasis (1), liver metastasis (1), and bone metastasis (3)). Group B had 2 deaths (9.1%) due to distant metastasis (survival times were 18 months and 24 months). 2 patients (9.1%) developed local recurrence (in-field GTV), 1 patient (4.5%) had regional recurrence, and 3 patients (13.6%) developed distant metastases (lung metastasis (1) and bone metastasis (2)). In group C, 1 patient (4.8%) died from distant metastasis (survival time was 35 months). All other patients in group C were free of local and regional recurrence. The 3-year LPF survival rates of three groups were 83.3%, 90.9% and 100%, respectively. The 3-year regional progression-free survival (RPFS) rates were 95.8%, 95.5% and 100%, respectively. The 3-year disease-free survival (DFS) rates were 79.2%, 86.4% and 95.2%, respectively. The 3-year OS rates were 83.3%, 90.9% and 95.2%, respectively. When comparing Group C with group A, 3-year LPF and DFS were statistically significant (P <0.05) (Fig 2). It is important to note that none of the patients who did not receive adjuvant chemotherapy had recurrent disease. Complete data is included in S2 Table.

### Toxicity

Toxicity results are shown in Table 3 (acute) and Table 4 (late). The most common acute toxicity was mucositis, with 52.4% to 54.6% grade 2 toxicity and 28.6% to 31.8% grade 3 toxicity. Late toxicities were grade 1–2 skin dystrophy, subcutaneous fibrosis, xerostomia, and hearing loss. There were no grade 4 late toxicities. No temporal lobe necrosis was observed. There was no significant difference in the acute radiation reactions among three groups.

**Table 3:** The frequency of acute toxicities for the three groups [No. of patient (%)].

	Grade 0	Grade 1	Grade 2	Grade 3	χ^2^ value	*P* value
Mucositis						
Group A	0 (0)	3 (12.5)	13 (54.2)	8 (33.3)		
Group B	0 (0)	3 (13.6)	12 (54.6)	7 (31.8)		
Group C	0 (0)	4 (19.0)	11 (52.4)	6 (28.6)	0.452	0.978
Nausea/vomiting						
Group A	1 (4.2)	10 (41.7)	13 (54.2)	0 (0)		
Group B	2 (9.1)	9 (40.9)	11 (50.0)	0 (0)		
Group C	2 (9.5)	13 (61.9)	6 (28.6)	0 (0)	3.668	0.453
Bone marrow suppression						
Group A	5 (20.8)	9 (37.5)	7 (29.2)	3 (12.5)		
Group B	6(27.3)	8 (36.3)	6 (27.3)	2 (9.1)		
Group C	4(19.0)	9 (42.9)	7 (33.3)	1 (4.8)	1.386	0.967
Skin desquamation						
Group A	4 (16.7)	15 (62.5)	3 (12.5)	2 (8.3)		
Group B	3 (13.6)	15 (68.2)	3 (13.6)	1 (4.6)		
Group C	4 (19.0)	14 (66.7)	2 (9.5)	1 (4.8)	0.767	0.993

**Table 4:** The frequency of late toxicities for the three groups [No. of patient (%)].

	Group A	Group B	Group C	*P* value
Xerostomia				
0	4(16.7)	4(18.2)	5(23.8)	0.968
1	13(54.2)	11(50.0)	11(52.4)	
2	7(29.1)	7(31.8)	5(23.8)	
3	0 (0)	0 (0)	0 (0)	
Skin fibrosis				
0	14(58.3)	13(59.1)	15(71.4)	0.942
1	8(33.3)	7(31.8)	5(23.8)	
2	2(8.3)	2(9.1)	1(4.8)	
3	0 (0)	0 (0)	0 (0)	
Subcutaneous fibrosis				
0	3(12.5)	2(9.1)	4(19.0)	0.962
1	13(54.2)	13(59.1)	11(52.4)	
2	8(33.3)	7(31.8)	6(28.6)	
3	0 (0)	0 (0)	0 (0)	
Hearing loss				
0	3(12.5)	4(18.2)	5(23.8)	0.932
1	14(58.3)	13(59.1)	11(52.4)	
2	7(29.2)	5(22.7)	5(23.8)	
3	0 (0)	0 (0)	0 (0)	

## Discussion

The treatment of locally advanced NPC remains challenging. Despite the advent of concurrent chemoradiotherapy and IMRT delivery techniques, patients still develop local recurrence within GTV. Multiple reports [4–8] have shown that local control of NPC is correlated with radiotherapy dose; therefore, many studies have evaluated radiotherapy dose-escalation in NPC. However, these dose-escalation studies have been single-arm without comparison to conventional radiotherapy. Moreover, dose-escalation has been associated with significant toxicities. For example, Bakst RL et al. at Memorial-Sloan Kettering Cancer Center (MSKCC) reported that there is an increased incidence of in-field brain radiation necrosis with dose-escalation in their prospective dose-escalation trial for nasopharyngeal carcinoma [9]. One strategy to accomplish dose-escalation while minimizing toxicity is to improve the delineation of the tumor volume that needs high-dose radiotherapy. In general, the GTV (area that needs high dose radiotherapy) in radiotherapy is defined by CT imaging. We hypothesized that we can improve the gross tumor delineation in nasopharyngeal carcinoma by utilizing PET/CT. This study aimed to compare conventional radiotherapy to CT-guided dose escalated radiotherapy and PET/CT-guided dose escalated radiotherapy in a randomized fashion.

There are several methods to dose-escalate GTV in NPC. One of the more effective techniques is the SMART-IMRT technique. It delivers both accelerated radiotherapy and hypofractionated radiotherapy to the GTV. Such an approach can significantly increase the biologically effective dose (BED) delivered. For example, the BED to GTV is 87 Gy in group B and 95 Gy in group C. However, the difference in BED between groups A and B is not significant (84 Gy vs 87 Gy). Compared to A, Group B is a more hypofractionated regimen which can have consequences to normal tissue toxicity. Based on the effective dose-escalation with SMART, we utilized this radiotherapy technique in our trial.

Despite the frequent utilization of PET/CT in radiotherapy in head and neck cancer, no study has directly compared PET/CT-guided dose-escalation to CT-guided dose-escalation in NPC. Our study aimed to be the first to directly compare the two techniques. We demonstrated that 47.6% (10/21) of the patients had significant changes in GTV volume with PET/CT planning when compared to that of CT-based planning. Our results confirmed that PET/CT can be helpful in tumor volume determination. Our result is not surprising, as Paulino et al. has shown that GTV determined using PET/CT can be significantly different from that of CT-based planning [15].

Our study showed that the 3-year local PFS rates of the three treatment groups were 83.3%, 90.9% and 100%, respectively. The difference of PFS between groups A and C is statistically significant, though the differences between groups A and B and B and C are not. The 3-year DFS rates were 79.2%, 86.4% and 95.2%, respectively. Although distant metastasis was the major cause of death in patients after treatment, our results suggests that the risk of distant metastasis was decreased with the increase of local control rates. However, it is important to note the limitation of our study. Our data was generated from a single institution pilot trial and the study may be under-powered. Our data is hypothesis generating and should be further validated in a large multi-center randomized trial.

The most common acute toxicities were acute mucositis, incidence rates of grade 2 ranged from 52.4% to 54.6%, and grade 3 ranged from 28.6% to 31.8%. No grade 4 late toxicities were noted. There was no significant difference in the acute radiation reactions among three groups. Late toxicities were grade 1–2 skin dystrophy, subcutaneous fibrosis, xerostomia, and hearing loss. The results of toxicities were similar to with conventional radiotherapy. We also did not observe any radiation brain necrosis, even in the CT-guided dose-escalation group. The difference in brain toxicities between our trial and the MSKCC trial is likely due to the fact that our CT-guided dose-escalation group was treated to a lower BED dose. Our PET/CT-guided dose-escalation group in general had smaller GTV volumes, which can explain the lack of brain toxicity in this group.

## Conclusions

PET-CT fusion may have significant impact on staging and radiotherapy treatment delineation in NPC. PET/CT-guided dose escalation radiotherapy appears to be well-tolerated. The SMART-IMRT technique to enhance BED of GTV, combined with concurrent chemotherapy, is completely feasible for local advanced NPC. Although the technical advancements have made it possible to use PET-CT in the radiotherapy planning process, it needs to be supported by robust clinical data in future studies.

## Supporting Information

S1 Checklist CONSORT checklist. (DOC)

S1 Protocol Study protocol. (DOC)

S1 Table Patient information and data. (XLS)

S2 Table PET/CT data. (XLS)
