# Supplementary material for: A Randomized Pilot Trial Comparing Position Emission Tomography (PET)-Guided Dose Escalation Radiotherapy to Conventional Radiotherapy in Chemoradiotherapy Treatment of Locally Advanced Nasopharyngeal Carcinoma
Source: PLoS One. 2015 Apr 27;10(4):e0124018. doi: 10.1371/journal.pone.0124018 (PMC4411028; doi:10.1371/journal.pone.0124018)
Supplement: S1 Checklist — (DOC) [file pone.0124018.s001.doc]

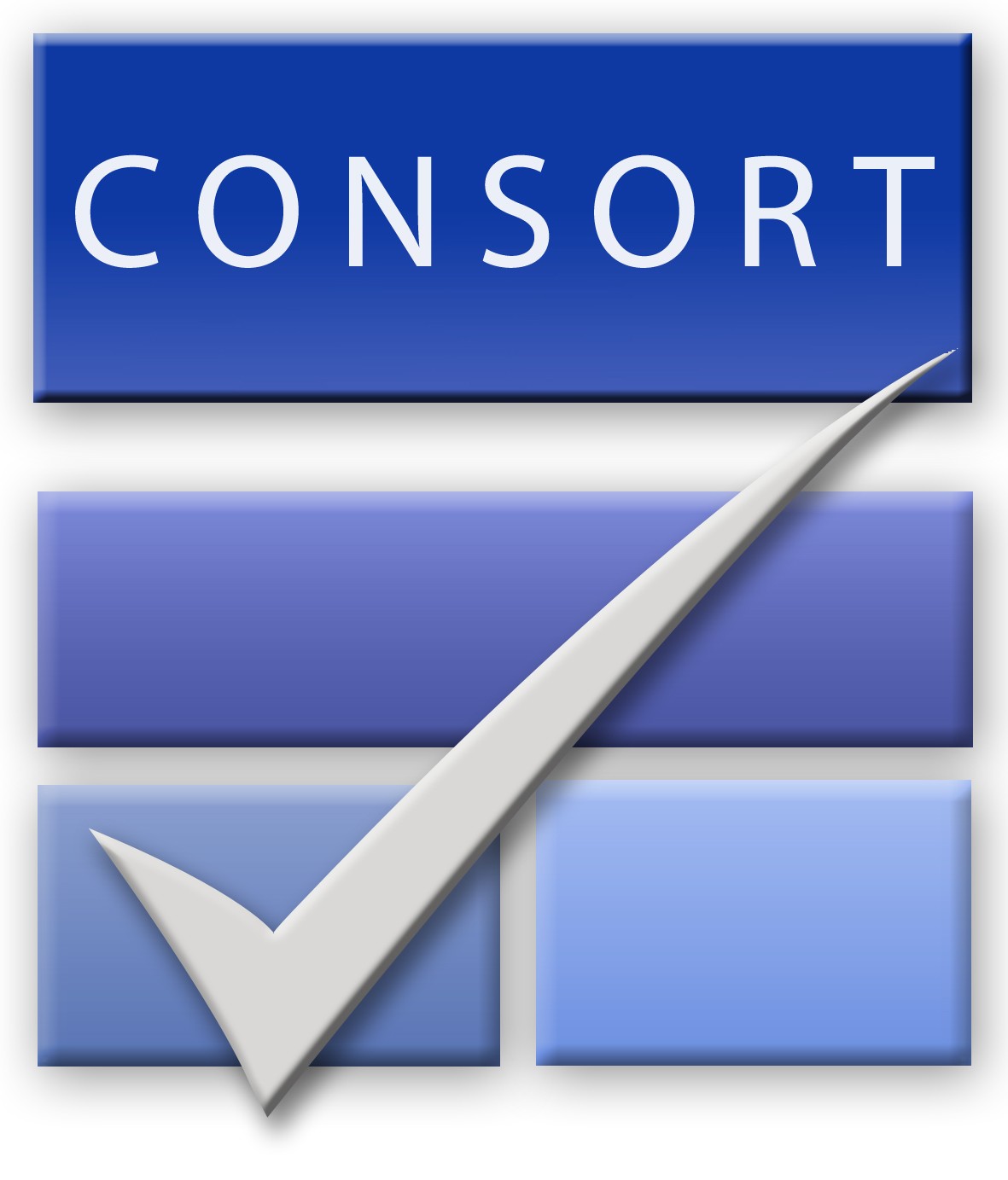
CONSORT 2010 checklist of information to include when reporting a randomised trial*

| Section/Topic | Item No | Checklist item | Reported on section |
| --- | --- | --- | --- |
| Title and abstract | | | |
|  | 1a | Identification as a randomised trial in the title | Title |
|  | 1b | Structured summary of trial design, methods, results, and conclusions (for specific guidance see CONSORT for abstracts) | Abstract |
| Introduction | | | |
| Background and objectives | 2a | Scientific background and explanation of rationale | Introduction |
|  | 2b | Specific objectives or hypotheses | Introduction |
| Methods | | | |
| Trial design | 3a | Description of trial design (such as parallel, factorial) including allocation ratio | Methods and S1 Protocol |
|  | 3b | Important changes to methods after trial commencement (such as eligibility criteria), with reasons | S1 Protocol |
| Participants | 4a | Eligibility criteria for participants | Methods and S1 Protocol |
|  | 4b | Settings and locations where the data were collected | Methods and S1 Protocol |
| Interventions | 5 | The interventions for each group with sufficient details to allow replication, including how and when they were actually administered | Methods and S1 Protocol |
| Outcomes | 6a | Completely defined pre-specified primary and secondary outcome measures, including how and when they were assessed | Methods and S1 Protocol |
|  | 6b | Any changes to trial outcomes after the trial commenced, with reasons | N/A |
| Sample size | 7a | How sample size was determined | Methods and S1 Protocol |
|  | 7b | When applicable, explanation of any interim analyses and stopping guidelines | N/A |
| Randomisation: |  |  |  |
| Sequence generation | 8a | Method used to generate the random allocation sequence | Methods and S1 Protocol |
|  | 8b | Type of randomisation; details of any restriction (such as blocking and block size) | Methods and S1 Protocol |
| Allocation concealment mechanism | 9 | Mechanism used to implement the random allocation sequence (such as sequentially numbered containers), describing any steps taken to conceal the sequence until interventions were assigned | Methods and S1 Protocol |
| Implementation | 10 | Who generated the random allocation sequence, who enrolled participants, and who assigned participants to interventions | S1 Protocol |
| Blinding | 11a | If done, who was blinded after assignment to interventions (for example, participants, care providers, those assessing outcomes) and how | N/A |
|  | 11b | If relevant, description of the similarity of interventions | N/A |
| Statistical methods | 12a | Statistical methods used to compare groups for primary and secondary outcomes | Methods and S1 Protocol |
|  | 12b | Methods for additional analyses, such as subgroup analyses and adjusted analyses | Methods and S1 Protocol |
| Results | | | |
| Participant flow (a diagram is strongly recommended) | 13a | For each group, the numbers of participants who were randomly assigned, received intended treatment, and were analysed for the primary outcome | Results |
|  | 13b | For each group, losses and exclusions after randomisation, together with reasons | S1 Table |
| Recruitment | 14a | Dates defining the periods of recruitment and follow-up | Results |
|  | 14b | Why the trial ended or was stopped | Results |
| Baseline data | 15 | A table showing baseline demographic and clinical characteristics for each group | Table 1 |
| Numbers analysed | 16 | For each group, number of participants (denominator) included in each analysis and whether the analysis was by original assigned groups | Results |
| Outcomes and estimation | 17a | For each primary and secondary outcome, results for each group, and the estimated effect size and its precision (such as 95% confidence interval) | Results |
|  | 17b | For binary outcomes, presentation of both absolute and relative effect sizes is recommended | Results |
| Ancillary analyses | 18 | Results of any other analyses performed, including subgroup analyses and adjusted analyses, distinguishing pre-specified from exploratory | Results |
| Harms | 19 | All important harms or unintended effects in each group (for specific guidance see CONSORT for harms) | Results |
| Discussion | | | |
| Limitations | 20 | Trial limitations, addressing sources of potential bias, imprecision, and, if relevant, multiplicity of analyses | Discussion |
| Generalisability | 21 | Generalisability (external validity, applicability) of the trial findings | Discussion |
| Interpretation | 22 | Interpretation consistent with results, balancing benefits and harms, and considering other relevant evidence | Discussion |
| Other information | | |  |
| Registration | 23 | Registration number and name of trial registry | Abstract |
| Protocol | 24 | Where the full trial protocol can be accessed, if available | S1 Protocol |
| Funding | 25 | Sources of funding and other support (such as supply of drugs), role of funders | Funding |

*We strongly recommend reading this statement in conjunction with the CONSORT 2010 Explanation and Elaboration for important clarifications on all the items. If relevant, we also recommend reading CONSORT extensions for cluster randomised trials, non-inferiority and equivalence trials, non-pharmacological treatments, herbal interventions, and pragmatic trials. Additional extensions are forthcoming: for those and for up to date references relevant to this checklist, see [www.consort-statement.org](http://www.consort-statement.org/).

**Checklist items**

**Title and abstract**

*Item 1a. Identification as a randomised trial in the title*

PET/CT guided radiotherapy, randomised clinical trial of eﬃcacy and safety.

*Item 1b. Structured summary of trial design, methods, results, and conclusions (for specific guidance see CONSORT for abstracts)*

**See** **Table 1**

**Table 1 Items to include when reporting a randomised trial in a journal abstract**

| Item | Description |
| --- | --- |
| Authors | Longzhen Zhang, MD and PhD |
| Trial design | Case-Control, Prospective |
| Methods: |  |
| Participants | Patients with previously untreated Stages III and IVA (AJCC 6th Edition) of locally advanced NPC, and histologically confirmed NPC by biopsy, no evidence of distant metastasis, Karnofsky performance status≥70, and good bone marrow, liver and kidney function (white blood cell count ≥ 4.0×10^9^/L, platelets ≥ 100×10^9^/L, albumin ≥30 g/L, creatinine ≤100μmol/L). Ages eligible for study: 18 yeas to 70 years. Patients those with a prior (within 5 years) or synchronous malignancy were excluded. |
| Interventions | conventional-IMRT: contrast-enhanced CT; SMART-IMRT: contrast-enhanced CT; SMART-IMRT: PET-CT. |
| Objective | Our study aims to compare the local control, overall survival and toxicities of PET-guided dose escalation radiotherapy and conventional radiotherapy in chemoradiotherapy treatment of locally advanced nasopharyngeal carcinoma. |
| Outcome | PET/CT-guided dose escalation radiotherapy is well-tolerated and appears to be superior to conventional chemoradiotherapy for locally advanced NPC |
| Randomisation | This test uses a computer-generated random number table randomization. Patients who met the eligibility criteria were randomized 1:1:1 into the three treatment arms |
| Blinding(masking) | single-blinded |
| Results: |  |
| Numbers randomised | 85 patients |
| Recruitment | 126 patients |
| Numbers analysed | 67 patients |
| Outcome | For the primary outcome, a result for each group and the estimated effect size and its precision were correct |
| Harms | The most common acute toxicity was mucositis, with 52.4% to 54.6% grade 2 toxicity and 28.6% to 31.8% grade 3 toxicity. Late toxicities were grade 1-2 skin dystrophy, subcutaneous fibrosis, xerostomia, and hearing loss. There were no grade 4 late toxicities. |
| Conclusions | PET-CT fusion may have significant impact on staging and radiotherapy treatment delineation in NPC. PET/CT-guided dose escalation radiotherapy appears to be well-tolerated. The SMART-IMRT technique to enhance BED of GTV, combined with concurrent chemotherapy, is completely feasible for local advanced NPC. |
| Trial registration | The trial is registered at ClinicalTrials.gov, number NCT02089204. |
| Funding | This study was supported by grants from the National Natural Science Foundation of China (No.81071831), Jiangsu Provincial Health Bureau issues (NO.H201021) and Xuzhou City Science and Technology Bureau issues (NO. XF10C082). |

**Introduction**

*Item 2a. Scientific background and explanation of rationale*

Nasopharyngeal carcinoma (NPC) differs from other head and neck malignancies in terms of its epidemiology, pathology, and treatment outcomes. It is endemic in China and is one of the major public health problems. Concurrent radiotherapy and chemotherapy is the primary treatment for patients with NPC. Despite such aggressive treatment, many patients with locally advanced NPC still develop locally recurrent disease. Since local control is directly related to patient morbidity and mortality in NPC, there is a strong need to identify methods to further improve treatment outcome for NPC.

One strategy to improve local control is to escalate the dose of radiotherapy. This is because local control has been shown to be directly related to the radiotherapy dose. Several different techniques, including brachytherapy, stereotactic radiosurgery, and dose-painting intensity modulated radiotherapy (IMRT), have been used to increase radiotherapy dose. However, due to the large number of critical anatomic structures near the nasopharynx, dose-escalation in NPC can also lead to increased toxicities. The main challenge for such a treatment is to identify the appropriate tumor volume to receive the high-dose radiotherapy. Conventional dose-escalation is conducted using computed tomography (CT) to identify the gross tumor volume (GTV). However, recent progress with ﬂuorine-18-ﬂuorode-oxyglucose positron emission tomography/computed tomography (^18^F-FDG-PET/CT) in treatment planning allows more accurate tumor volume delineation. We hypothesize that the use of PET/CT in treatment planning can improve dose-escalation radiotherapy for NPC, which in turn can improve therapeutic efficacy while reducing toxicity. Given that there has been no clinical trials directly comparing conventional chemoradiotherapy to CT-guided dose-escalation chemoradiotherapy or PET/CT guided dose-escalation chemoradiotherapy in locally advanced NPC, our study aims to compare the local control, overall survival and toxicities of the three treatment regimens.

*Item 2b. Specific objectives or hypotheses*

PET-CT fusion may have significant impact on staging and radiotherapy treatment delineation in NPC. PET/CT-guided dose escalation radiotherapy appears to be well-tolerated. The SMART-IMRT technique to enhance BED of GTV, combined with concurrent chemotherapy, is completely feasible for local advanced NPC.

**Methods**

*Item 3a. Description of trial design (such as parallel, factorial) including allocation ratio*

This was a single center, prospective, stratiﬁed (SMART-IMRT, PET-CT, with randomisation [1:1:1]), single-blinded, case -controlled, parallel-group study conducted in the China.

*Item 3b. Important changes to methods after trial commencement (such as eligibility criteria), with reasons*

From June 2012, PET/CT-guided dose escalation radiotherapy become two groups: ^18^F-MISO PET/CT-guided dose escalation chemoradiotherapy and ^18^F-FDG PET/CT-guided dose escalation chemoradiotherapy. We wanted to see whether hypoxia imaging-guided dose escalation radiotherapy improve the efficacy of Nasopharyngeal Carcinoma.

*Item 4a. Eligibility criteria for participants*

Patients with previously untreated Stages III and IVA (AJCC 6th Edition) of locally advanced NPC, and histologically confirmed NPC by biopsy, no evidence of distant metastasis, Karnofsky performance status≥70, and good bone marrow, liver and kidney function (white blood cell count ≥ 4.0×10^9^/L, platelets ≥ 100×10^9^/L, albumin ≥30 g/L, creatinine ≤100μmol/L). Ages eligible for study: 18 yeas to 70 years. Patients those with a prior (within 5 years) or synchronous malignancy were excluded.

*Item 4b. Settings and locations where the data were collected*

The study took place at the Department of Radiation Oncology, Affiliated Hospital of Xuzhou Medical College, China, from February 2009 to April 2014.

*Item 5. The interventions for each group with* *sufﬁcient details to allow replication, including how and when they were actually administered*

For radiotherapy treatments, In the groups A and B, GTVs were delineated based on fusing diagnostic CT images with simulation CT images. For group C, images from a diagnostic PET/CT are fused to the treatment CT. Patients in group A received radiotherapy delivered with the intensity modulated radiotherapy (IMRT) technique. PTV_1_ received 70 Gy in 2 Gy per fraction whereas PTV_2_ received 58 Gy in 2 Gy per fraction in groups A. For groups B and C, PTV_1_ received 64 Gy and PTV_2_ received 54 Gy, all in 1.8 Gy per fraction. In addition, the GTV received 70 Gy in 2.18 Gy per fraction in group B and the GTV received 77Gy in 2.34 Gy per fraction in group C. Radiotherapy was delivered using the SMART-IMRT technique in the dose-escalation treatment arms. Parotid gland dose was limited to a mean dose below 30Gy and the temporal lobes dose was limited to below 55Gy, both without compromising PTV or GTV coverage.

Concurrent chemotherapy consisted of cisplatin (20mg / m^2^, iv, d1- 4) and docetaxel (75mg / m^2^, d1, d8) administered on the 1st and 4th week of treatment. All patients received adjuvant chemotherapy that ranged from 2 to 4 cycles.

*Item 6a.* *Completely deﬁned pre-speciﬁed primary and secondary outcome measures, including how and when they were assessed*

The primary endpoint: 3-year local progression-free survival (LPF）

Secondary endpoints: 3-year disease-free survival (DFS), 3-year overall survival (OS), Short and long term toxicity, Quality of life of patients (QOL) analysis.

All primary and secondary outcome measures have completely defined.

*Item 6b. Any changes to trial outcomes after the trial commenced, with reasons*

No

*Item 7a. How sample size was determined*

To detect 3-year local progression-free survival (LPF） with a two-sided 5% significance level and a power of 80%, a sample size of 50 patients per group was necessary, given an anticipated dropout rate of 10%. To recruit this number of patients a 24-month inclusion period was anticipated. In this trial, because of poor recruitment, the actual sample size differed from the originally intended sample size. 30 patients per group, interim analyses are used to help decide whether to stop early or to continue recruiting sometimes beyond the planned trial end.

*Item 7b. When applicable, explanation of any interim analyses and stopping guidelines*

Two interim analyses was performed during the trial. The levels of significance maintained an overall P value of 0.05 and were calculated according to the O’Brien-Fleming stopping boundaries. Two formal interim analyses of efficacy were performed when 50% and 75% of the expected number of primary events had accrued; no correction of the reported P value for these interim tests was performed.

An independent data and safety monitoring board periodically reviewed the efficacy and safety data. We and a data monitoring committee took multiple “looks” at the data every six months, with the intention that the trial will cease if the observed P value is smaller than the critical value.

*Item 8a. Method used to generate the random allocation sequence*

For allocation of the participants, a computer-generated list of random numbers was used.

*Item 8b. Type of randomisation; details of any restriction (such as blocking and block size)*

Participants were randomly assigned following simple randomization procedures (computerized random numbers) into 3 treatment groups, with a 1:1:1 allocation using random block sizes.

*Item 9. Mechanism used to implement the random allocation sequence (such as sequentially numbered containers), describing any steps taken to conceal the sequence until interventions were assigned*

Three treatment methods were put into envelopes in advance and consecutively numbered. Patients who met the eligibility criteria were assigned an order number according to the randomisation schedule and received the envelopes in the corresponding number.

The allocation sequence was concealed from the researcher enrolling and assessing participants in sequentially numbered, opaque, sealed and stapled envelopes. Aluminium foil inside the envelope was used to render the envelope impermeable to intense light. To prevent subversion of the allocation sequence, the name and date of birth of the participant was written on the envelope. Corresponding envelopes were opened only after the enrolled participants completed all baseline assessments and it was time to allocate the intervention.

*Item 10. Who generated the allocation sequence, who enrolled participants, and who assigned participants to interventions*

Block randomisation was by a computer generated random number list prepared by an investigator with no clinical involvement in the trial. We stratified by admission for an oncology related procedure. After the research nurse had obtained the patient’s consent, she telephoned a contact who was independent of the recruitment process for allocation consignment.

Item 11a. If done, who was blinded after assignment to interventions (for example, participants, care providers, those assessing outcomes) and how

Whereas patients and physicians allocated to the intervention group were aware of the allocated arm, outcome assessors and data analysts were kept blinded to the allocation.

*Item 11b. If relevant, description of the similarity of interventions*

Three treatments were taking concurrent chemoradiotherapy, similar to the treatment process, and the same chemotherapy drugs.

*Item 12a. Statistical methods used to compare groups for primary and secondary outcomes*

The primary endpoint was 3-year local progression-free survival (LPF）of NPC. Secondary efficacy endpoints included 3-year disease-free survival (DFS), 3-year overall survival (OS), short and long term toxicity, Quality of life of patients (QOL) analysis. The Kaplan-Meier method was used to calculate the actuarial rates of local control, DFS and OS. The χ^2^ test was used to compare incidence rates and categorical variables and Student’s t-test was used to compare the means of continuous variables.

*Item 12b. Methods for additional analyses, such as subgroup analyses and adjusted analyses*

Proportions of patients responding were compared between treatment groups with the Mantel-Haenszel χ^2^ test

**Results**

*Item 13.* *Participant ﬂow (a diagram is strongly recommended)*

*Item 13a. For each group, the numbers of participants who were randomly assigned, received intended treatment, and were analysed for the primary outcome*

**See Fig 1**.

*Item 13b. For each group, losses and exclusions after randomisation, together with reasons*

There were seven patients losses and exclusions after randomisation.  They were not receive allocated intervention, due to concomitant health problem or not interested.

*Item 14a. Dates defining the periods of recruitment and follow-up*

Age-eligible participants were recruited in Affiliated Hospital of Xuzhou Medical College from February 2009 to March 2011. Participants attended clinic visits at the time of randomisation (base-line) and at 3-month intervals for 3 years.

*Item 14b. Why the trial ended or was stopped*

In the interim analysis found that PET / CT guided concurrent chemoradiotherapy group has better curative effect than other groups, all patients were recruited into the proceeding as planned, there was no end of the trial or stop

Lost to follow-up (n=4)

Lost to follow-up (n=4)

Allocated to intervention **PET/CT-SMART** (n=27)

Received allocated intervention (n=25)

Allocated to intervention **CT**- **SMART** (n=28)

Received allocated intervention (n=25)

Assessed for eligibility (n= 126)

Excluded (n=41)

 Not meeting inclusion criteria (n=32 )

 Declined to participate (n=9)

 Other reasons (n= 0 )

Randomized (n=85)

Allocated to intervention **CT-Uniform dose IMRT** (n=30)

Received allocated intervention (n=28)

Lost to follow-up (n=3)

Analysed (n=24)

Analysed (n=21)

Analysed (n=22)

**Fig 1** Flow diagram of a randomized pilot trial comparing position emission tomography (PET)-guided dose escalation radiotherapy to conventional radiotherapy in chemoradiotherapy treatment of locally advanced nasopharyngeal carcinoma. The diagram includes detailed information on the excluded participants.

*Item 15. A table showing baseline demographic and clinical characteristics for each group*

**See table 2**

*Item 16. For each group, number of participants (denominator) included in each analysis and whether the analysis was by original assigned groups*

The primary analysis was intention-to-treat and involved all patients who were randomly assigned. 4 patients in the group A were lost to follow up; thus data from 24 patients were available for the intention-to-treat analysis. In group B, 3 patients lost to follow up, 22 patients remained for the per-protocol analyses. In group C, 4 patients lost to follow up, 21 patients remained for the per-protocol analyses.

**Table 2.**  **The Demographic and Clinical Characteristics of 67 Patients**

| **Patient characteristics** | **Group A** | **Group B** | **Group C** | ***P*value*** |
| --- | --- | --- | --- | --- |
| Gender |  |  |  |  |
| Male | 15 | 14 | 14 |  |
| Female | 9 | 8 | 7 | ＞0.05 |
| Age (yr) |  |  |  |  |
| Range | 19~67 | 20~68 | 19~64 |  |
| Mean | 47 | 48 | 46 | ＞0.05 |
| [Clinical stages](dict://key.0895DFE8DB67F9409DB285590D870EDD/clinical stages) |  |  |  |  |
| Ⅲ stage | 14 | 13 | 14 |  |
| Ⅳa stage | 10 | 9 | 7 | ＞0.05 |
| T stage |  |  |  |  |
| T1 | 1 | 1 | 1 |  |
| T2 | 10 | 9 | 7 |  |
| T3 | 7 | 5 | 9 |  |
| T4 | 6 | 6 | 4 | ＞0.05 |
| N stage |  |  |  |  |
| N0 | 1 | 1 | 1 |  |
| N1 | 3 | 4 | 3 |  |
| N2 | 15 | 14 | 13 |  |
| N3 | 5 | 3 | 4 | ＞0.05 |
| Pathologic types |  |  |  |  |
| WHO II | 5 | 4 | 3 |  |
| WHO Ⅲ | 19 | 18 | 18 | ＞0.05 |

*Chi-square test performed.

Abbreviation: Group A: conventional chemoradiotherapy group; Group B: CT-guided dose escalation chemoradiotherapy group; Group C: PET/CT-guided dose escalation chemoradiotherapy group; WHO: World Health Organization.

*Item 17a. For each primary and secondary outcome, results for each group, and the estimated effect size and its precision (such as 95% conﬁdence interval)*

**See table 3**

*Item 17b. For binary outcomes, presentation of both absolute and relative effect sizes is recommended*

The risk of 3-year LPF was reduced by 25% (95% CI 31% to 14%), and the absolute difference was −17% (95% CI −25% to −8%). PET/CT-guided dose escalation radiotherapy could reduce 3-year LPF of locally advanced NPC.**See table 4**

Table 4 Reporting of both absolute and relative effect sizes

| **Primary endpoint** | **Group A** | **Group C** | **χ^2^ value** | ***P*value** | **Risk difference (95% CI)** |
| --- | --- | --- | --- | --- | --- |
| 3-year LPF | 83.3(20/24) | 100(21/21) | 3.391 | 0.046 | -17%(-25 to -8) |

Table 3 Reporting of summary results for each study group (%)

| **Endpoin** | **Group A** | **Group B** | **Group C** | **χ^2^ value** | ***P*value** | **Risk difference (95% CI)** |
| --- | --- | --- | --- | --- | --- | --- |
| **Primary endpoint** |  |  |  |  |  |  |
| 3-year LPF | 83.3(20/24) **^*^** | 90.9(20/22) | 100(21/21) **^*^** | 4.318 | 0.115 | 17%(5-26) |
| **Secondary endpoint** |  |  |  |  |  |  |
| 3-year RPF | 95.8(23/24) | 95.5(21/22) | 100(21/21) | 1.500 | 0.221 | 5%(1-10) |
| 3-year DFS | 79.2(19/24)^◢^ | 86.4(19/22) | 95.2(20/21) ^◢^ | 4.105 | 0.128 | 16%(5-26) |
| 3-year OS | 87.5(21/24) | 95.5(21/22) | 100(21/21) | 3.574 | 0.167 | 13%(4-24) |

*Item 18. Results of any other analyses performed, including subgroup analyses and adjusted analyses, distinguishing prespeciﬁed from exploratory*

One distinct advantage of PET/CT in radiotherapy planning is its potential to improve target identification, reducing intra-observer and inter-observer variability. We hypothesize that the use of PET/CT in treatment planning can improve dose-escalation radiotherapy for NPC which in turn can improve therapeutic efficacy while reducing toxicity. We prespecified our primary subgroup analysis on the basis of PET / CT positioning and SMART-IMRT. We also did prespeciﬁed secondary subgroup analyses based on sex, age, and c[linical stages](dict://key.0895DFE8DB67F9409DB285590D870EDD/clinical stages). For all subgroup analyses, we used the Kaplan-Meier method to calculate the actuarial rates of local control, DFS and OS. The χ2 test was used for comparing incidence rates and categorical variables and Student’s t-test was used for comparing the means of continuous variables, designated to be significant at p<0.05. Figure 2 shows the results of our prespecified subgroup analyses and indicates consistency of effects. Our subgroup analyses were underpowered to detect the modest differences in subgroup effects that one might expect to detect if there was a true subgroup effect.

*Item 19. All important harms or unintended effects in each group (For speciﬁc guidance see CONSORT for harms)*

Toxicity results are shown in Table 5. The most common acute toxicities were mucositis, with 52.4% to 54.6% grade 2 toxicity and 28.6%~31.8% grade 3 toxicity. Late toxicities were grade 1-2 skin dystrophy, subcutaneous fibrosis, xerostomia, and hearing loss. No patient had grade 4 late toxicity. All patients were not found in the temporal lobe damage in the follow-up period. There was no significant difference in the acute radiation reactions among three groups.

Table 5. The frequency of acute toxicities for the three groups [case (%)]

|  | Grade 0 | | Grade 1 | | Grade 2 | | Grade 3 | χ^2^ value | | *P* value |
| --- | --- | --- | --- | --- | --- | --- | --- | --- | --- | --- |
| Catarrh |  |  | |  | |  | |  |  | |
| Group A | 0 (0) | 3 (12.5) | | 13 (54.2) | | 8 (33.3) | |  |  | |
| Group B | 0 (0) | 3 (13.6) | | 12 (54.6) | | 7 (31.8) | |  |  | |
| Group C | 0 (0) | 4 (19.0) | | 11 (52.4) | | 6 (28.6) | | 0.452 | 0.978 | |
| Nausea/vomiting |  |  | |  | |  | |  |  | |
| Group A | 1 (4.2) | 10 (41.7) | | 13 (54.2) | | 0 (0) | |  |  | |
| Group B | 2 (9.1) | 9 (40.9) | | 11 (50.0) | | 0 (0) | |  |  | |
| Group C | 2 (9.5) | 13 (61.9) | | 6 (28.6) | | 0 (0) | | 3.668 | 0.453 | |
| Arrest of bone marrow |  |  | |  | |  | |  |  | |
| Group A | 5 (20.8) | 9 (37.5) | | 7 (29.2) | | 3 (12.5) | |  |  | |
| Group B | 6(27.3) | 8 (36.3) | | 6 (27.3) | | 2 (9.1) | |  |  | |
| Group C | 4(19.0) | 9 (42.9) | | 7 (33.3) | | 1 (4.8) | | 1.386 | 0.967 | |
| Skin desquamation |  |  | |  | |  | |  |  | |
| Group A | 4 (16.7) | 15 (62.5) | | 3 (12.5) | | 2 (8.3) | |  |  | |
| Group B | 3 (13.6) | 15 (68.2) | | 3 (13.6) | | 1 (4.6) | |  |  | |
| Group C | 4 (19.0) | 14 (66.7) | | 2 (9.5) | | 1 (4.8) | | 0.767 | 0.993 | |

**Discussion**

*Item 20. Trial limitations, addressing sources of potential bias, imprecision, and, if relevant, multiplicity of analyses*

Fewer patients enrolled were a limitation of our study. Because we was a single-center clinical trial, so patients enrolled more slowly. According to the current results of clinical studies, we decided to carry out the next multi-center collaboration to expand the number of samples.

*Item 21. Generalisability (external validity, applicability) of the trial ﬁndings*

PET-CT fusion may have a significant impact on staging and determination of RT treatment GTV volume and dose in NPC. PET/CT-guided dose escalation radiotherapy appears to be well-tolerated. SMART-IMRT technique to enhance BED of GTV and combined with concurrent chemotherapy is completely feasible for local advanced NPC. Although the technical advancements have made it possible to use PET-CT in the radiotherapy planning process, it needs to be supported by robust clinical data in future.

*Item 22. Interpretation consistent with results, balancing beneﬁts and harms, and considering other relevant evidence*

Given that there has been no clinical trials directly comparing conventional chemoradiotherapy to CT-guided dose-escalation chemoradiotherapy or PET/CT guided dose-escalation chemoradiotherapy in locally advanced NPC, our study aims to compare the local control, overall survival and toxicities of the three treatment regimens. In this single-center, randomised controlled trial, PET/CT-guided dose escalation radiotherapy is well-tolerated and appears to be superior to conventional chemoradiotherapy for locally advanced NPC.

**Other information**

*Item 23. Registration number and name of trial registry*

The trial is registered at ClinicalTrials.gov, number NCT02089204.

*Item 24. Where the full trial protocol can be accessed, if available*

Full details of the trial protocol can be found in the Supplementary Appendix, available with the full text of this article at *https://clinicaltrials.gov.*

*Item 25. Sources of funding and other support (such as supply of drugs), role of funders*

This study was supported by grants from the National Natural Science Foundation of China (No.81071831)，Jiangsu Provincial Health Bureau issues (NO.H201021) and Xuzhou City Science and Technology Bureau issues (NO. XF10C082).
