# Supplementary material for: A Randomized Pilot Trial Comparing Position Emission Tomography (PET)-Guided Dose Escalation Radiotherapy to Conventional Radiotherapy in Chemoradiotherapy Treatment of Locally Advanced Nasopharyngeal Carcinoma
Source: PLoS One. 2015 Apr 27;10(4):e0124018. doi: 10.1371/journal.pone.0124018 (PMC4411028; doi:10.1371/journal.pone.0124018)
Supplement: S1 Protocol — (DOC) [file pone.0124018.s002.doc]

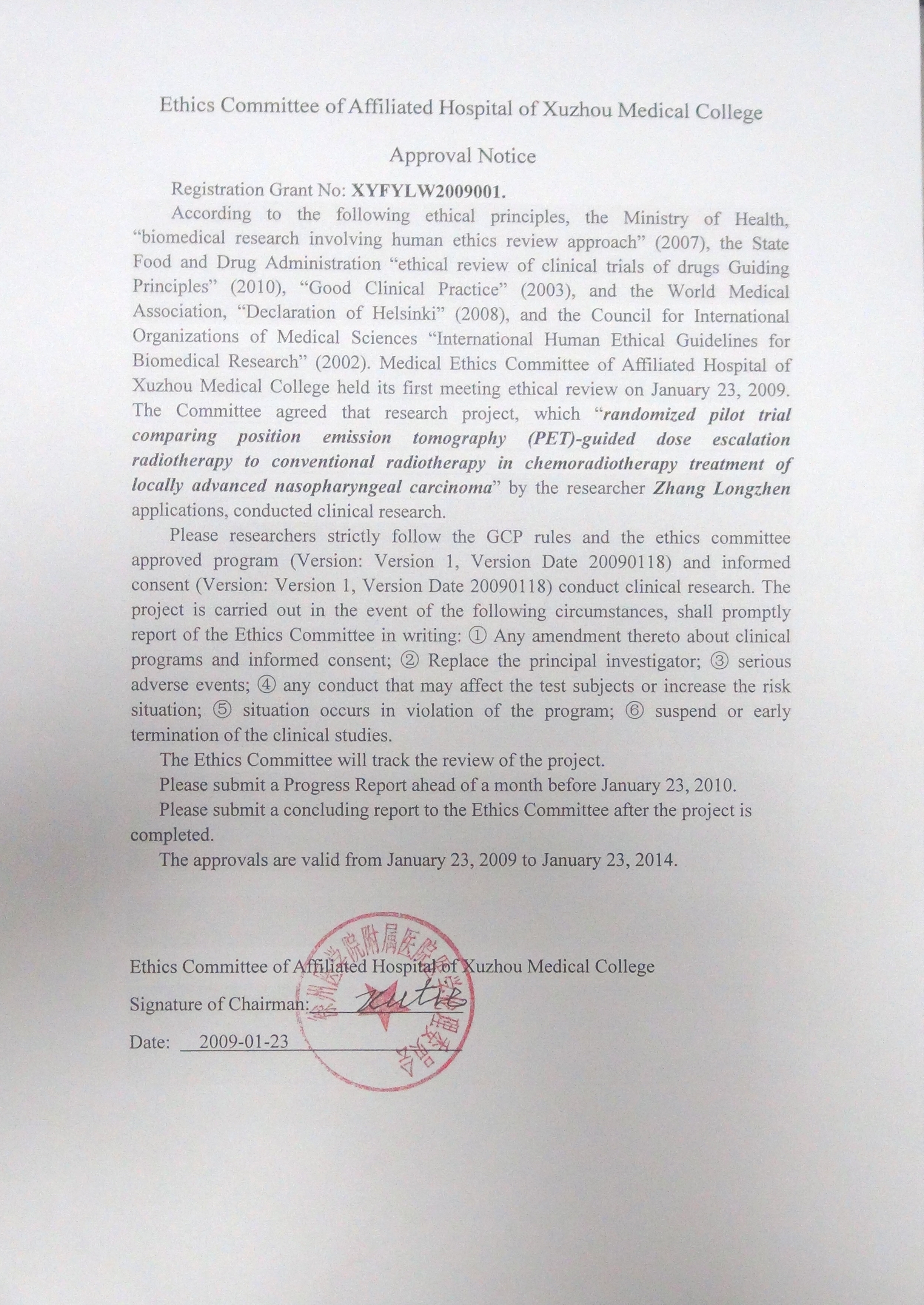


Affiliated Hospital of Xuzhou Medical College Clinical Research Ethics Review Application Form

**PARTⅠ: OUTLINE OF APPLICATION**

**﹡Mandatory field must be filled in. Put down “NA” if not applicable.**

1. Name of Study

| 1.1 | Scientific title (*should include study design, name of intervention, condition being studied and study outcomes*) | |
| --- | --- | --- |
|  | ﹡A randomized pilot trial comparing position emission tomography (PET)-guided dose escalation radiotherapy to conventional radiotherapy in chemoradiotherapy treatment of locally advanced nasopharyngeal carcinoma | |
| 1.1.1 | Research protocol number | ﹡XYFYLW2009001 |
| 1.2 | Short Title (for lay public / easy quote) | |
|  | ﹡PET/CT guided radiotherapy | |

**2. Applicant (Principal Investigator)**

| 2.1 | Title: | ﹡MD and Chief Physician | | | | Surname: | | ﹡Zhang | First name: | | ﹡Longzhen |  |
| --- | --- | --- | --- | --- | --- | --- | --- | --- | --- | --- | --- | --- |
|  | Name in Chinese: | | | | | ﹡章龙珍 | | | | | |  |
| 2.1.1 | Staff: ﹡ | | | | | | | | | | |  |
|  | × University staff | | | | Position: | | Xuzhou Medical College | | | | |  |
|  | ×Affiliated Hospital staff | | | | Position: | | Affiliated Hospital of Xuzhou Medical College | | | | |  |
|  | HA staff | | | | Position: | |  | | | | |  |
|  | Department / Unit: | | | | School of Medical Imaging  Department of Radiation Oncology | | | | | | |  |
| 2.1.2 | For student project: ﹡ | | | | | | | | | | |  |
|  | Full-time University student | | | | | | Undergraduate University student | | | | |  |
|  | Part-time University student | | | | | | Post-graduate University student | | | | | |
|  | Name of Programme (Submit supporting document): | | | | | |  | | | | | |
|  | Name of academic supervisor: | | |  | | | School / Faculty: | | |  | | |
|  | Name of site supervisor: | | |  | | | Department/ Unit: | | |  | | |
| 2.2 | The PI’s primary affiliated institution/hospital: ﹡ | | | | | | | | | | | |
|  | Department of Radiation Oncology, Affiliated Hospital of Xuzhou Medical College; Cancer Institute of Xuzhou Medical College, Jiangsu, China. | | | | | | | | | | | |
| 2.3 | Qualifications and relevant experience ( Please add details in your Short CV if there is not enough space) | | | | | | | | | | | |
|  | MD, chief physician , professor, master tutor Oncology, Director of Radiation Oncology, Affiliated Hospital of Xuzhou Medical College. Chaired three provincial and municipal scientific research , one National Natural Science Foundation. She has published more than 40 papers. | | | | | | | | | | | |
| 2.4 | Phone number: | | +86 18052268368 | | | | | | | | | |
| 2.5 | Fax number: | | - | | | | | | | | | |
| 2.6 | E-mail: | | jsxzzlz@126.com | | | | | | | | | |
| 2.7 | Mailing address | | No. 99 Huaihai West Road, Quanshan District, Xuzhou, Jiangsu, China. PC:221002 | | | | | | | | | |

**3. Co-investigators (Short CV for all Co-investigators should be submitted.)**

| Surname | First name | Title | Relevant qualifications | Department | Institution/Hospital |
| --- | --- | --- | --- | --- | --- |
| Zhang | Longzhen | Chief Physician | MD, Professor | Radiation Oncology | Affiliated Hospital of Xuzhou Medical College |
| Wang | Jianshe | Physician |  | Radiation Oncology | Affiliated Hospital of Xuzhou Medical College |
| Xin | Yong | Physician |  | Radiation Oncology | Affiliated Hospital of Xuzhou Medical College |
| Tang | Tianyou | Deputy Chief Physician |  | Radiation Oncology | Affiliated Hospital of Xuzhou Medical College |
| Lu | Cunzhi | Deputy Chief Physician |  | Nuclear Medicine | PET-CT Center Xuzhou Central Hospital |
| Chen | Yong | Competent technician |  | Radiation Oncology | Affiliated Hospital of Xuzhou Medical College |
| Wu | Yang | Residency |  | Radiation Oncology | Affiliated Hospital of Xuzhou Medical College |
| Yan | Honglian | Graduate |  | Radiation Oncology | Xuzhou Medical College |
|  |  |  |  |  |  |

**4. Study Site(s) {Fill in Item 4.3.2 if PI conducts study in other site(s).}**

| 4.1 | Is this a local or international trial? | | ﹡Local |
| --- | --- | --- | --- |
| 4.2 | Is there a plan to involve more than one HA cluster? | | ﹡NO |
| 4.3 | Study sites in China | Department | Hospital(fill in more rows if necessary) |
| 4.3.1 | Applying site﹡ | Department of Radiation Oncology | Affiliated Hospital of Xuzhou Medical College |
| 4.3.2 | Collaborating site(s) ﹡ | Nuclear Medicine | PET-CT Center Xuzhou Central Hospital |
|  |  |
|  |  |
|  |  |

**5. Parallel Ethics Review for Cross-cluster Study**

| 5.1 | Has the protocol been reviewed by another Cluster REC/IRB? | N/A(not planned for Cross-cluster involvement) |
| --- | --- | --- |
|  | **(If yes, please state the Cluster REC/IRB in Item 5.1.1 in supplementary sheet)** | |
| 5.1.1 | What is the REC decision?(Provide proof if available) | Don’t know |

**6. Milestones**

| 6.1 | Proposed study start date: | 2 / 2009 (mm / yyyy) ﹡ |
| --- | --- | --- |
| 6.2 | Proposed study end date or date of last follow-up of all recruited subjects, whichever is later: | 4 / 2014 (mm / yyyy) ﹡ |
| 6.3 | Expected final report date to Cluster REC: | 4 / 2014 (mm / yyyy) ﹡ |

**7. Brief Summary of Study** (＜500 words, use language that can be understood by laypersons)

| This pilot trial is designed to determine whether PET/CT-guided radiotherapy dose escalation can improve local control while minimizing toxicity for the treatment of locally advanced nasopharyngeal carcinoma. One distinct advantage of PET/CT in radiotherapy planning is its potential to improve target identification, reducing intra-observer and inter-observer variability. We hypothesize that the use of PET/CT in treatment planning can improve dose-escalation radiotherapy for NPC which in turn can improve therapeutic efficacy while reducing toxicity. We prespecified our primary subgroup analysis on the basis of PET / CT positioning and SMART-IMRT. We also did prespeciﬁed secondary subgroup analyses based on sex, age, and c[linical stages](dict://key.0895DFE8DB67F9409DB285590D870EDD/clinical stages). For all subgroup analyses, we used the Kaplan-Meier method to calculate the actuarial rates of local control, DFS and OS. The χ2 test was used for comparing incidence rates and categorical variables and Student’s t-test was used for comparing the means of continuous variables, designated to be significant at p<0.05. PET-CT fusion may have significant impact on staging and radiotherapy treatment delineation in NPC. PET/CT-guided dose escalation radiotherapy appears to be well-tolerated. The SMART-IMRT technique to enhance BED of GTV, combined with concurrent chemotherapy, is completely feasible for local advanced NPC. |
| --- |

**8. Major Ethical Issues** (＜500 words, use language that can be understood by laypersons)

| We considered that the use of 18F-FDG-PET/CT may slight impact on the surrounding environment, especially in the patient's excreta.  Control Measures: All patients were required to sign an informed consent form before proceeding to clinical trials. Patient’s PET/CT scanning in strict accordance with PET/CT examination processes. Patients need to wait to check in a separate waiting rooms or wards in the hospital setting PET-CT centers. All patient’s excrement (feces, urine, etc.) were separately destroyed. Patients were isolated for observation to a specified time after the inspection is completed. given radiation dose detection before return to the general ward. |
| --- |

**PART Ⅱ: STUDY DETAILS**

**9.** Scientific basis

| 9.1 | Background, current evidence and key references: | | |
| --- | --- | --- | --- |
|  | Nasopharyngeal carcinoma (NPC) differs from other head and neck malignancies in terms of its epidemiology, pathology, and treatment outcomes. It is endemic in China and is one of the major public health problems. Concurrent radiotherapy and chemotherapy is the primary treatment for patients with NPC. In recent years, with the development and application of comprehensive treatment and Intensity Modulated Radiation Therapy (IMRT) technology, the overall prognosis of nasopharyngeal carcinoma has been significantly improved, 5-year survival rate was 74.5% , 3 -year local control rate was 90%^[2]^. Despite such aggressive treatment, many patients with locally advanced NPC still develop locally recurrent disease ^[3]^.Since local control is directly related to patient’s morbidity and mortality in NPC, there is a strong requirement to develop a method for improving current treatments’ efficacy.  One strategy to improve local control is escalating the dose of radiotherapy. This is because local control has been shown to be directly related to the radiotherapy dose . Several techniques, including brachytherapy , stereotactic radiosurgery , and dose-painting intensity modulated radiotherapy (IMRT) , have been used to increase radiotherapy dose. However, due to the large number of critical anatomic structures near the nasopharynx, dose-escalation in NPC can also lead to increased toxicities . One of the more effective techniques is the simultaneous modulated accelerated radiation therapy (SMART) IMRT technique ^[10]^. It delivers both accelerated radiotherapy and hypofractionated radiotherapy to the GTV. The main challenge for these treatments is to identify the appropriate tumor volume that could be received the high-dose radiotherapy. Conventional dose-escalation is conducted via using computed tomography (CT) to identify the gross tumor volume (GTV). However, recent progress in the field of ﬂuorine-18-ﬂuorode-oxyglucose positron emission tomography/computed tomography (18F-FDG-PET/CT)-adjuvant treatment planning allows more accurate tumor volume delineation ]. Thus, we hypothesize that of 18F-FDG-PET/CT-adjuvant treatment planning can improve dose-escalation radiotherapy for NPC, which in turn can improve therapeutic efficacy as well as reducing toxicity.  To identify this hypothesis, we compared the efficacy and toxicity of three treatments, including conventional chemoradiotherapy, CT-guided dose-escalation chemoradiotherapy and PET/CT guided dose-escalation chemoradiotherapy, in patients with locally advanced NPC via a randomized clinical trial in our department. PET-CT fusion may have significant impact on staging and radiotherapy treatment delineation in NPC. PET/CT-guided dose escalation radiotherapy appears to be well-tolerated. The SMART-IMRT technique to enhance BED of GTV, combined with concurrent chemotherapy, is completely feasible for local advanced NPC.  [1] Wei WI, Sham JS. Nasopharyngeal carcinoma. Lancet. Jun 11-17 2005;365(9476):2 041-2054.  [2] Lu H, Peng L, Yuan X, Hao Y, et al.Concurrent chemoradiotherapy in locally advanced nasopharyngeal carcinoma: a treatment paradigm also applicable to patients in Southeast Asia. Cancer Treat Rev. 2009, 35 (4):345-353.  [3] Wee J, Tan EH, Tai BC, et al. Randomized trial of radiotherapy versus concurrent chemoradiotherapy followed by adjuvant chemotherapy in patients with American Joint Committee on Cancer/International Union against cancer stage III and IV nasopharyngeal cancer of the endemic variety. J Clin Oncol. 2005;23(27):6730-6738.  [4] Marks JE, Bedwinek JM, Lee F, Purdy JA, Perez CA. Dose-response analysis for nasopharyngeal carcinoma: an historical perspective. Cancer. Sep 15 1982;50(6):1042-1050.  [5] Teo PM, Leung SF, Tung SY, et al. Dose-response relationship of nasopharyngeal carcinoma above conventional tumoricidal level: a study by the Hong Kong nasopharyngeal carcinoma study group (HKNPCSG). Radiotherapy and oncology : journal of the European Society for Therapeutic Radiology and Oncology. Apr 2006;79(1):27-33.  [6] Teo PM, Leung SF, Lee WY, Zee B. Intracavitary brachytherapy significantly enhances local control of early T-stage nasopharyngeal carcinoma: the existence of a dose-tumor-control relationship above conventional tumoricidal dose. International journal of radiation oncology, biology, physics. Jan 15 2000;46(2):445-458.  [7] Hara W, Loo BW, Jr., Goffinet DR, et al. Excellent local control with stereotactic radiotherapy boost after external beam radiotherapy in patients with nasopharyngeal carcinoma. International journal of radiation oncology, biology, physics. Jun 1 2008;71(2):393-400.  [8] Lin S, Pan J, Han L, et al. Nasopharyngeal carcinoma treated with reduced-volume intensity-modulated radiation therapy: report on the 3-year outcome of a prospective series. Int J Radiat Oncol Biol Phys. 2009; 75(4):1071-8.  [9] Ashamalla H, Guirgius A, Bieniek E, et al. The impact of positron emission tomography/computed tomography in edge delineation of gross tumor volume for head and neck cancers [J]. Int J Radiat Oncol Biol Phy, 2007, 68 (2):388-395.  [10] Butler EB, Teh BS, Grant WH, 3rd, et al. Smart (simultaneous modulated accelerated radiation therapy) boost: a new accelerated fractionation schedule for the treatment of head and neck cancer with intensity modulated radiotherapy. International journal of radiation oncology, biology, physics. Aug 1 1999;45(1):21-32.  [11] Madani I, Duthoy W, Derie C, et al. Positron emission tomography-guided, focal-dose escalation using intensity-modulated radiotherapy for head and neck cancer. International journal of radiation oncology, biology, physics. May 1 2007;68(1):126-135.  . | | |
| 9.2 | Aim of study: | | |
|  | This pilot trial is designed to determine whether PET/CT-guided radiotherapy dose escalation can improve local control while minimizing toxicity for the treatment of locally advanced nasopharyngeal carcinoma. | | |
| 9.3 | Hypothesis (e.g. *Compared to x control, y intervention leads to a greater rate of z outcome.*) | | |
|  | PET/CT-guided dose escalation radiotherapy is well-tolerated for patients with locally advanced NPC as compared to conventional chemoradiotherapy. | | |
| 9.4 | Primary outcome(s) | Outcome measure(s) | Time-point |
|  |  | Local progression-free survival (LPF） | 3-year |
|  |  |  |  |
| 9.5 | Secondary outcome(s) | Disease-free survival (DFS) | 3-year |
|  |  | Overall survival (OS) | 3-year |
|  |  | Short term toxicity | 3-month |
|  |  | Long term toxicity | 3-year |
|  |  |  |  |
| 9.6 | In what way will the research contribute to knowledge or healthcare development? | | |
|  | The project findings will be published in the form of paper, to be published 2-4 papers. The results of this study will be used on the clinical. | | |

**10. Study subjects**

| 10.1 | Inclusion criteria: |
| --- | --- |
|  | Patients with previously untreated Stages III and IVA (AJCC 6th Edition) of locally advanced NPC, and histologically confirmed NPC by biopsy, no evidence of distant metastasis, Karnofsky performance status≥70, and good bone marrow, liver and kidney function (white blood cell count ≥ 4.0×10^9^/L, platelets ≥ 100×109/L, albumin ≥30 g/L, creatinine ≤100μmol/L). Ages eligible for study: 18 yeas to 70 years. Patients those with a prior (within 5 years) or synchronous malignancy were excluded. |
| 10.2 | Exclusion criteria: |
|  | There was evidence of distant metastases, previous malignancy or other concomitant malignant disease, pregnant or lactating, nasopharyngeal radiotherapy history, patients withdrew during treatment or doctor and other factors caused by violation of the study protocol. |
| 10.3 | Sample-size and rationale for calculation: |
|  | Sample-size= 90 ﹡  Based on the following rationale: To detect 3-year local progression-free survival (LPF） with a two-sided 5% significance level and a power of 80%, a sample size of 30 patients per group was necessary, given an anticipated dropout rate of 10%. |
| 10.4 | Number of subjects to be recruited locally in relation to this application: |
|  | n= 90 ﹡in applying site. |
| 10.5 | How will subjects to be identified and recruited? |
|  | We will publish the recruitment information of clinical trials on the website of Xuzhou Medical College Hospital (***http://www.jsxyfy.com/***). According to the specific inclusion criteria and exclusion criteria rigorous screening patients. |

**11. Risk Assessment whether Expedited Review is Suitable**

| 11.1 | Will study incur extra clinical intervention(s) to subjects? | | | | NO |
| --- | --- | --- | --- | --- | --- |
| 11.2 | Will study impose additional risk to subjects? | | | | NO |
| 11.3 | Will study raise sensitive / important privacy concerns? | | | | NO |
| 11.4 | Will the study involve the following vulnerable subjects? | | | |  |
| 11.4.1 | - Foetuses in Uteri/ non-viable foetuses / abortus | | | | NO |
| 11.4.2 | - Infants (age 0 to ＜1) | | | | NO |
| 11.4.3 | - Children (age 1 to ＜13) | | | | NO |
| 11.4.4 | - Adolescents (age 13 to ＜18) | | | | NO |
| 11.4.5 | - Pregnant / lactating women | | | | NO |
| 11.4.6 | - Persons related unequally to investigators, e.g. student, employee | | | | NO |
| 11.4.7 | - Special population, e.g. prisoner, mentally / cognitively disabled | | | | NO |
| 11.4.8 | - Others: | NO | Specify if yes: |  | |
| 11.5 | Are there any special precautions to protect the interest of vulnerable subjects? | | | | |
|  | In the course of treatment, when patients with adverse reactions, it will get timely medical assistance. According to the patient informed consent, waive part of medical expenses. | | | | |

**12. Study Design and Methodology**

| 12.1 | Study design: | | Prospective ﹡; and Randomized controlled trial ﹡ | | | | | | | |
| --- | --- | --- | --- | --- | --- | --- | --- | --- | --- | --- |
|  | If others, specify: | |  | | | | | | | |
| 12.1.1 | Methods of assignment: | | | Randomization | | | | | | |
| 12.1.2 | Control: | | | Concurrent control (cohort) | | | | | | |
| 12.1.3 | Degree of masking: | | | Single-blinded | | | | | | |
| 12.1.4 | Group assignment: | | | Parallel | | | | | | |
| 12.1.5 | Phase of study: | | | Phase Ⅱ | | | | | | |
| **For PhaseⅠStudy, please complete the “Supplementary Information Sheet for PhaseⅠStudy ”.** | | | | | | | | | | |
| 12.2 | Disease group (*choose the most appropriate one*) | | | | | ENT system | | | | |
| 12.2.1 | Key conditions under study *(e.g. asthma; DM etc)* | | | | | Nasopharyngeal carcinoma; SMART-IMRT; PET-CT; chemoradiotherapy | | | | |
| 12.3 | Study article (Please put “NA” on the field of “Generic name” if nor applicable) | | | | | | | | | |
| Article | Type﹡ | Generic name | | | Duration of exposure | | Dosage | Route of administration | | Was it produced under GMP? |
| 1 | Radiotherapy | SMART-IMRT;PET/CT | | | 7 Week(s) | | 77Gy | Others | | N/A |
| 2 | Radiotherapy | SMART-IMRT; CT | | | 7 Week(s) | | 70Gy | Others | | N/A |
|  |  |  | | |  | |  |  | |  |
|  |  |  | | |  | |  |  | |  |
| Control | Radiotherapy | IMRT; CT | | | 7 Week(s) | | 70Gy | Others | | N/A |
|  | | | | | | | | | | |
| 12.4 | Will an application of clinical trial certificate be made? | | | | | Yes | | | | |
| 12.5 | Has a PhaseⅠstudy been done? | | | | | Yes (for all study articles) | | | | |
| 12.6 | Number of extra visits / admissions on top of usual care: | | | | | | | | | |
|  | 10-20 patients in each group. | | | | | | | | | |
| 12.7 | Will any of the study interventions / procedures be performed by persons other than the investigators, and if so by whom and where? | | | | | | | | | |
|  | Block randomisation was by a computer generated random number list prepared by an investigator with no clinical involvement in the trial. We stratified by admission for an oncology related procedure. After the research nurse had obtained the patient’s consent, she telephoned a contact who was independent of the recruitment process for allocation consignment. | | | | | | | | | |
| 12.8 | Will biological samples or date be stored for future use? | | | | | | | | NO | |
| 12.8.1 | If yes, give details and explain how consent will be obtained : | | | | | | | | | |
|  |  | | | | | | | | | |

**13. Methods of Statistical Analysis**

| All events were measured from the date of randomization. OS was defined as the time from the date of radiotherapy to death or the latest date known to be alive. Durations were calculated from the end of treatment. The Kaplan-Meier method was used to calculate the actuarial rates of local control, DFS and OS. The χ2 test was used to compare incidence rates and categorical variables and Student’s t-test was used to compare the means of continuous variables, designated to be significant at p<0.05. |
| --- |

**14. Potential Risk Arising from Study**

| 14.1 | Induce discomfort or distress | Yes |
| --- | --- | --- |
| 14.2 | More invasive than the usual management | No﹡ |
| 14.3 | Increase physical or psychological risk | No |
| 14.4 | Involve a potential toxin, mutagen or teratogen | No |
| 14.5 | Involve radiation or radioactive substance | Yes |
| 14.6 | Incur other hazards | No |
| 14.7 | If yes to any of the above, provide details: | |
|  | Item 14.1.The most common acute toxicities were mucositis, with 52.4% to 54.6% grade 2 toxicity and 28.6%~31.8% grade 3 toxicity. Late toxicities were grade 1-2 skin dystrophy, subcutaneous fibrosis, xerostomia, and hearing loss. No patient had grade 4 late toxicity.  Item 14.5. Radiation therapy itself involves radiation. The use of 18F-FDG-PET/CT may slight impact on the surrounding environment, especially in the patient's excreta. | |
| 14.8 | Significant difference(s) from usual management: | |
|  | PET/CT-guided dose escalation radiotherapy is well-tolerated for patients with locally advanced NPC as compared to conventional chemoradiotherapy. | |

**15. Anticipated Benefits to Study Subjects**

| PET-CT fusion may have significant impact on staging and radiotherapy treatment delineation in NPC. PET/CT-guided dose escalation radiotherapy appears to be well-tolerated. The SMART-IMRT technique to enhance BED of GTV, combined with concurrent chemotherapy, is completely feasible for local advanced NPC. |
| --- |

**16. Research Subject Protection**

| 16.1 | Will subjects be provided with a card indicating their participation in study and means of urgent contact? | Yes |
| --- | --- | --- |
| 16.2 | Does protocol state compliance with the Declaration of Helsinki?  (http://www.wma.net/e/ethicsunit/helsinki.htm) | Yes |
| 16.3 | Does protocol state compliance with ICH-GCP? | Yes |

**17. Information and Consent**

| 17.1 | In what form will consent be obtained? | | | | | Written |
| --- | --- | --- | --- | --- | --- | --- |
| 17.1.1 | State reasons if not written or applying to waive the consent requirement | | | | | |
|  |  |  |  |  |  |  |
| 17.2 | Will an interpreter be available when required? | | | | | Yes |
| 17.3 | In obtaining informed consent from subjects, what is the minimal time given to a subject to consider after explanation has been given? | | | | | |
|  | 3 Day(s) | | | | | |
| 17.4 | Who will carry out the informed consent process? | | | | | |
|  | × | Principal investigator | × | Co-investigators | | |
|  |  | Research assistant |  | Others, specify: |  | |
| 17.5 | If subjects are incompetent in giving consent, to whom will the study be explained? | | | | | |
|  | Principal investigator | | | | | |

**18. Data and Safety Monitoring**

| 18.1 | Will an independent committee review data and safety of study? | | Yes local﹡ |
| --- | --- | --- | --- |
| 18.2 | If yes, who funds this committee? | Hospital and principal investigator | |
| 18.3 | Composition | Principal investigator | |

**19. Confidentiality and Use of Results**

| 19.1 | How will data be handled and stored during and after completion of the study, and who will be responsible for its safekeeping？ |
| --- | --- |
|  | The research center has a full-time staff for handling and storing data during the study. All data will be sent to archives of ethics committee for safekeeping after completion of the study. |
| 19.2 | Who will have access to the data or study record during or after the study? |
|  | Principal investigator and authorized personnel |
| 19.3 | How long will the data be kept and what will be done with them after completion of storage period? |
|  | 10 years |

**PART Ⅲ：BUDGET AND USE OF RESOURCES**

**20. Source of Funding**

| 20.1 | Commercial source: | No﹡ | Sponsor(if applicable): ﹡ |
| --- | --- | --- | --- |
|  |  | Name (1) |  |
|  |  | Name (2) |  |
| 20.2 | Non-  commercial source: | Type of funding | Granting body (if applicable): ﹡ |
|  |  | Local research grant﹡ | the National Natural Science Foundation of China (No.81071831) |
|  |  | Local research grant﹡ | Jiangsu Provincial Health Bureau issues (NO.H201021) |
|  |  | Local research grant﹡ | Xuzhou City Science and Technology Bureau issues (NO. XF10C082). |
|  |  |  |  |

**21. Resources Implication and Conflict of Interest**

| 21.1 | Will this study use HA resources? | | No﹡ |
| --- | --- | --- | --- |
| 21.1.1 | If yes, provide details: | | |
|  |  | | |
| 21.2 | How will this affect the waiting time of other patients with competing needs? | | |
|  | Potential biases do not affect results of waiting time study | | |
| 21.3 | Will study site (hospital) receive reimbursement for the study? | No﹡ | |
| 21.3.1 | Is there a non-monetary (drug, consumable, equipment or research assistant) sponsorship? | No﹡ | |
| 21.3.2 | If yes, give details: | | |
|  | | |

**22. Financial Costs and Payment to Subjects**

| 22.1 | Will the subjects be charged for the study article/service | No |
| --- | --- | --- |
| 22.2 | Will the study article continue to be available to subjects after study (if subjects benefited from it) until it is commercially available? | No |
| 22.3 | If yes to either 22.1/22.2, how will be the cost be met and by whom? | |
|  | |
| 22.4 | Does the consent form explain the above arrangement? | Yes |
| 22.5 | Will subjects receive payment? | No |
| 22.5.1 | If yes, specify nature, amount and payment schedule: | |
|  | |

**23. Research Organization and Indemnity**

| 23.1 | The organization/individual responsible for securing the arrangements to initiate and/or manage a study: | | |
| --- | --- | --- | --- |
|  | Department of Radiation Oncology, Affiliated Hospital of Xuzhou Medical College. Zhang longzhen, Professor and MD | | |
| 23.2 | Collaborating parties that jointly take on the responsibilities for the study: | | |
|  | Collaborating party(1) | Department of Radiation Oncology, University of North Carolina-Chapel Hill, Chapel Hill, NC, USA | |
|  | Collaborating party(2) | PET-CT Center, Xuzhou Central Hospital, Xuzhou, Jiangsu, China. | |
|  | Collaborating party(3) |  | |
| 23.3 | If this is an industry sponsored trial, will the sponsor indemnify study related claims? | | N/A﹡ |
|  | If yes, :answer the following | | |
| 23.3.1 | -Is the indemnity agreement based on the HA approved form? | | N/A |
| 23.3.2 | -Will a copy of the agreement be submitted to Cluster REC? | | N/A |
| 23.3.3 | -Will a copy of the insurance policy backing the indemnity be submitted to Cluster REC? | | N/A﹡ |


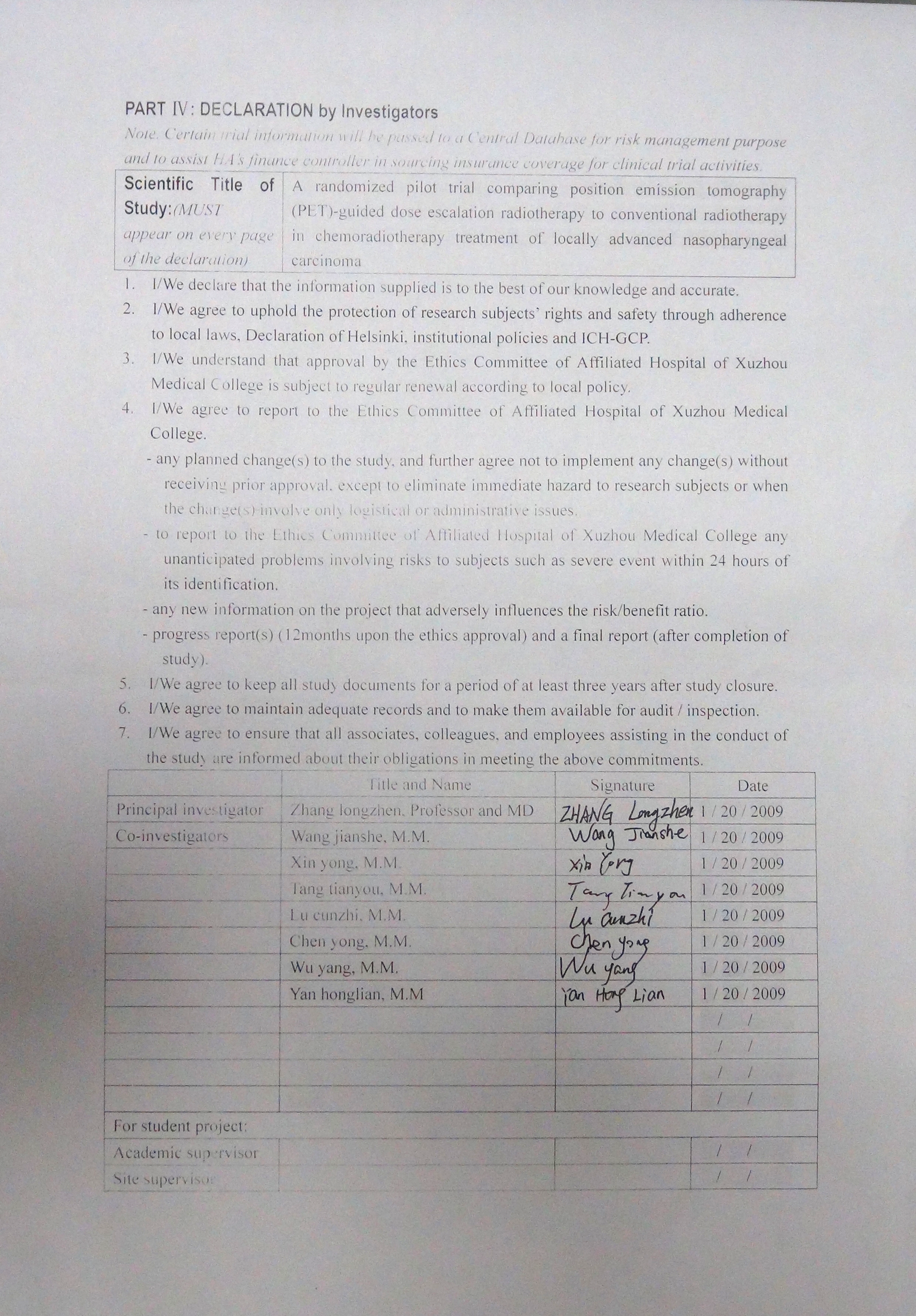
**PART Ⅳ: DECLARATION by Investigators**

*Note. Certain trial information will be passed to a Central Database for risk management purpose and to assist HA’s finance controller in sourcing insurance coverage for clinical trial activities.*

| **Scientific Title of Study:***(MUST appear on every page of the declaration)* | A randomized pilot trial comparing position emission tomography (PET)-guided dose escalation radiotherapy to conventional radiotherapy in chemoradiotherapy treatment of locally advanced nasopharyngeal carcinoma |
| --- | --- |

1. I/We declare that the information supplied is to the best of our knowledge and accurate.

2. I/We agree to uphold the protection of research subjects’ rights and safety through adherence to local laws, Declaration of Helsinki, institutional policies and ICH-GCP.

3. I/We understand that approval by the Ethics Committee of Affiliated Hospital of Xuzhou Medical College is subject to regular renewal according to local policy.

4. I/We agree to report to the Ethics Committee of Affiliated Hospital of Xuzhou Medical College.

- any planned change(s) to the study, and further agree not to implement any change(s) without receiving prior approval, except to eliminate immediate hazard to research subjects or when the change(s) involve only logistical or administrative issues.

- to report to the Ethics Committee of Affiliated Hospital of Xuzhou Medical College any unanticipated problems involving risks to subjects such as severe event within 24 hours of its identification.

- any new information on the project that adversely influences the risk/benefit ratio.

- progress report(s) (12months upon the ethics approval) and a final report (after completion of study).

5. I/We agree to keep all study documents for a period of at least three years after study closure.

6. I/We agree to maintain adequate records and to make them available for audit / inspection.

7. I/We agree to ensure that all associates, colleagues, and employees assisting in the conduct of the study are informed about their obligations in meeting the above commitments.

|  | Title and Name | Signature | Date |
| --- | --- | --- | --- |
| Principal investigator | Zhang longzhen, Professor and MD |  | 1 / 20 / 2009 |
| Co-investigators | Wang jianshe, M.M. |  | 1 / 20 / 2009 |
|  | Xin yong, M.M. |  | 1 / 20 / 2009 |
|  | Tang tianyou, M.M. |  | 1 / 20 / 2009 |
|  | Lu cunzhi, M.M. |  | 1 / 20 / 2009 |
|  | Chen yong, M.M. |  | 1 / 20 / 2009 |
|  | Wu yang, M.M. |  | 1 / 20 / 2009 |
|  | Yan honglian, M.M |  | 1 / 20 / 2009 |
|  |  |  | / / |
|  |  |  | / / |
|  |  |  | / / |
|  |  |  | / / |
| For student project: | | | |
| Academic supervisor |  |  | / / |
| Site supervisor |  |  | / / |
